# Supplementary material for: Alpha-Cyclodextrin Attenuates the Glycemic and Insulinemic Impact of White Bread in Healthy Male Volunteers
Source: Foods. 2020 Jan 7;9(1):62. doi: 10.3390/foods9010062 (PMC7023330; doi:10.3390/foods9010062)

APPENDIX A

Individual blood glucose and blood insulin levels after consumption of 100 g white bread and 250 mL water (red symbols) and 100 g white bread and 250 mL water with 25 g dissolved alpha-cyclodextrin (blue symbols).

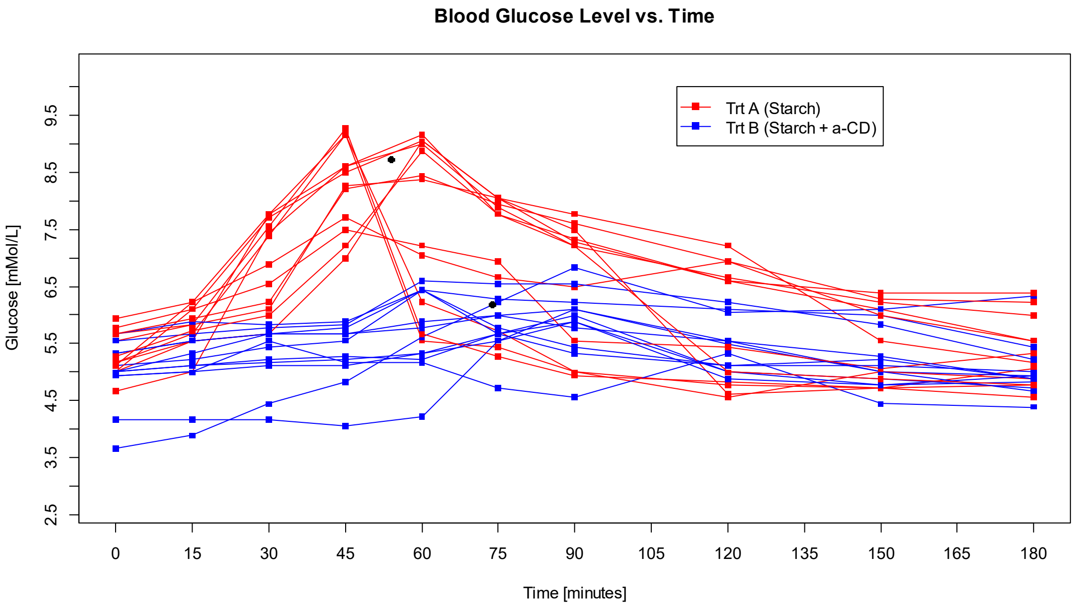

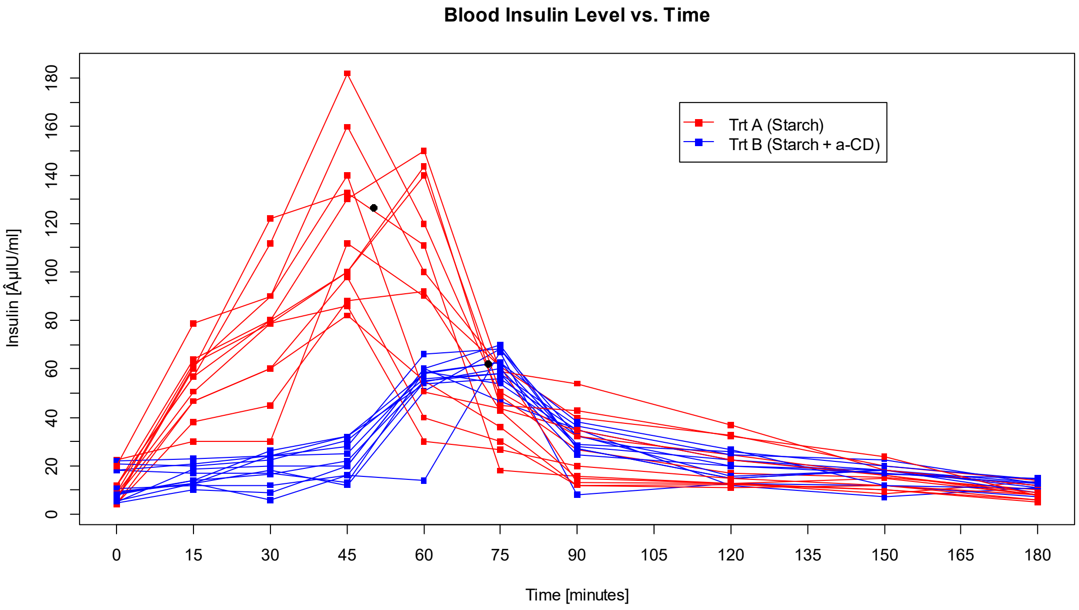

APPENDIX B

Mosaik plots of individual subjects: Blood glucose and insulin concentrations after ingestion of 100 g white bread consumed with 250 mL plain drinking water (treatment A) or 250 mL water containing 10 g dissolved alpha-cyclodextrin (treatment B) or 250 mL water with 25 g alpha-CD (treatment C). Numbers are the number of the subjects (n= 12 in total).

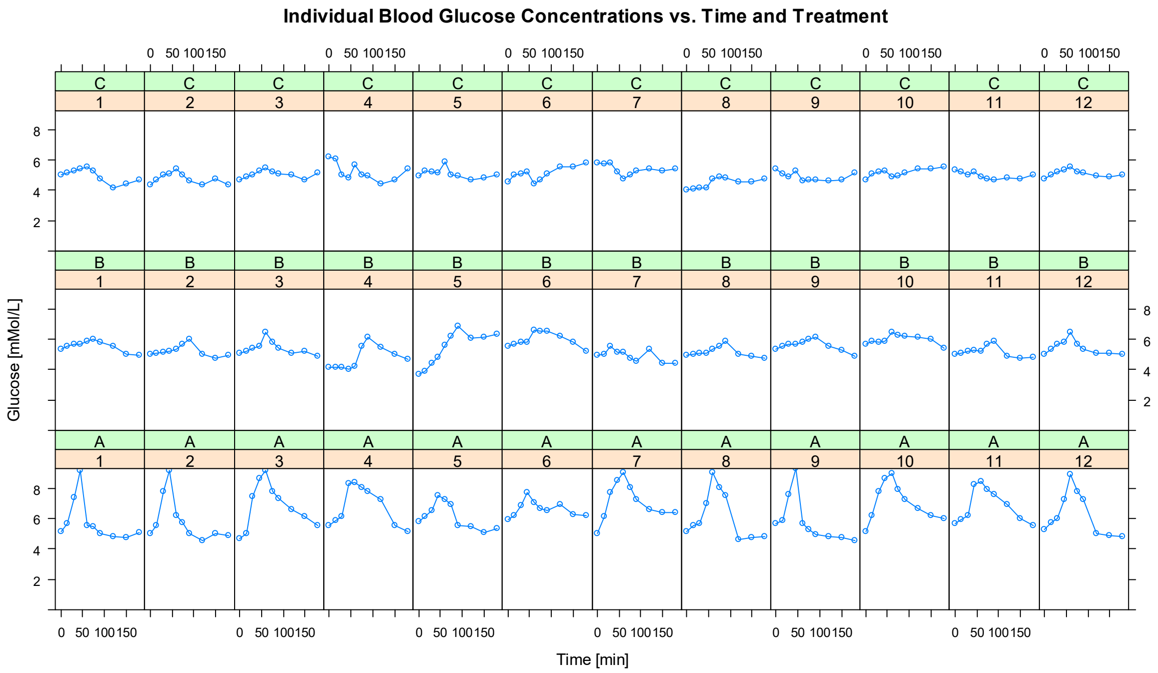

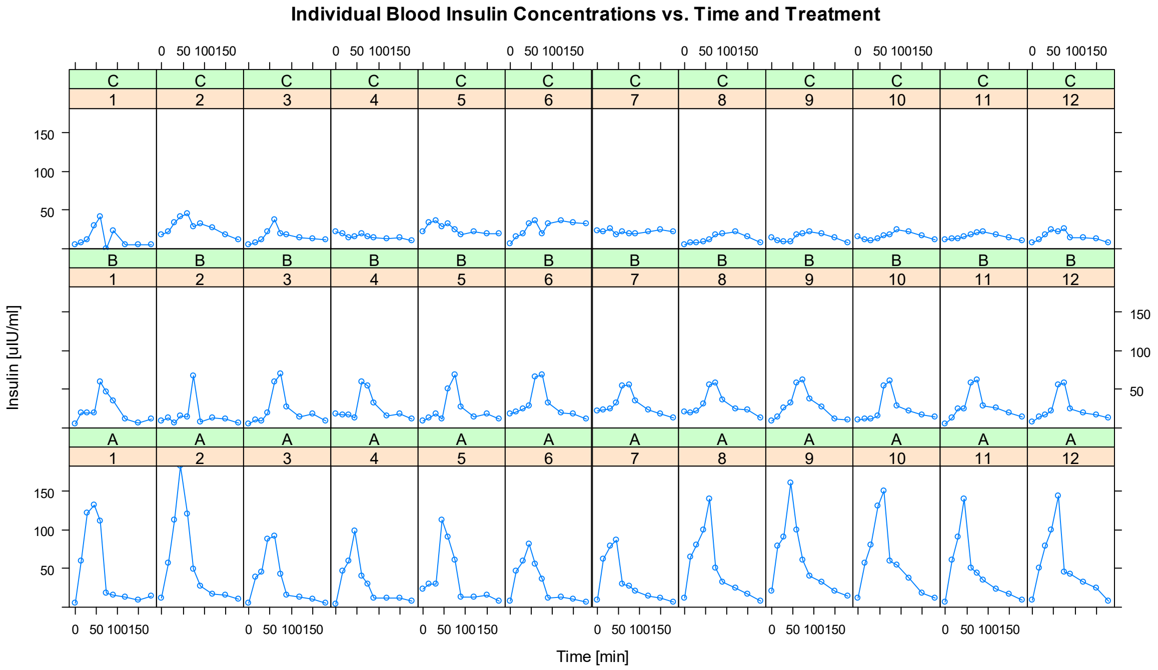

Supplement: Supplementary file 1 [file foods-09-00062-s001.pdf]
